# Supplementary material for: Mutualism increases diversity, stability, and function of multiplex networks that integrate pollinators into food webs
Source: Nat Commun. 2020 May 1;11:2182. doi: 10.1038/s41467-020-15688-w (PMC7195475; doi:10.1038/s41467-020-15688-w)
Supplement: Supplementary file 1 — Supplementary Information [file 41467_2020_15688_MOESM1_ESM.pdf]

Supplementary Information for

Mutualism increases diversity, stability, and function of multiplex networks that integrate pollinators into food webs

Kayla R. S. Hale*, Fernanda S. Valdovinos & Neo D. Martinez

*Correspondence to: Kayla R. S. Hale 1105 North University Ave, Biological Sciences Building, Ann Arbor, MI 48109. E-mail: kaylasal@umich.edu

Supplementary Information includes:

SUPPLEMENTARY METHODS
Plant-pollinator network construction.
Uncertainty and sensitivity analyses.

SUPPLEMENTARY DISCUSSION

Supplementary Table 1: Parameters and quantitative results of uncertainty analyses.
Supplementary Table 2: Parameters and quantitative results of sensitivity analyses.
Supplementary Table 3: Qualitative effects of variation in parameter values on persistence, abundance, and productivity.
Supplementary Table 4: Summary of symbols, definitions, and values used in the main text.

Supplementary Figure 1: Properties of empirical and simulated plant-pollinator networks.
Supplementary Figure 2: Persistence along a gradient of increasing floral rewards productivity.
Supplementary Figure 3: Effects of increasing mutualism on plant persistence.
Supplementary Figure 4: Robustness of diversity and persistence to simulation length.
Supplementary Figure 5: Robustness of abundance, function, and temporal stability to simulation length.
Supplementary Figure 6: Diversity and persistence in feedback controls.
Supplementary Figure 7: Abundance, function, and temporal stability in feedback controls.
Supplementary Figure 8: Composition changes in feedback controls.
Supplementary Figure 9: Example timeseries comparing multiplex and feedback control simulations.
Supplementary Figure 10: Connectance of network treatments.

SUPPLEMENTARY REFERENCES

SUPPLEMENTARY METHODS

Plant-pollinator network construction.

To synthesize network structure in our multiplex model, we attempted to create empirically-realistic food webs and plant-pollinator networks that could be interconnected. This required us to choose a common definition of nodes for these networks, as nodes in empirical plant-pollinator networks often represent taxonomic species while nodes in empirical food webs are often represented as 'trophic species,' in which all taxonomic species with similar sets of predators and prey are grouped into one node [1–3]. Because trophic-grouping can reduce methodological bias in empirical food web data and additionally facilitate comparison to structural models [4], we followed a similar approach to aggregate species in empirical plant-pollinator networks (N = 49 networks listed in Supplementary Table 1 of ref. [5]). Specifically, we grouped species with exactly the same set of mutualistic partners into one node. This tended to group super-generalist plants and specialist pollinators into single nodes, increasing connectance (C_p), reducing species diversity (S_p), and thus reducing the average ratio of animal to plant species (A/P) of empirical networks from 2.5 to 2 (Supplementary Fig. 1a). The 95% confidence intervals around the diversity-connectance relationship in the grouped empirical networks were defined by the following equations: $C_{p_{\min}} = 1.05811 S_p^{-0.6572}$, $C_{p_{\max}} = 2.1737 S_p^{-0.4779}$, where $C_{p_{\min}}$ bounds the minimum connectance and $C_{p_{\max}}$ bounds the maximum connectance across a range of diversities.

We generated plant-pollinator networks within this range of empirical properties using the Thébault and Fontaine's stochastic mutualistic network algorithm [6] (main text Fig. 2b). The algorithm takes as input the number of pollinators (A), the number of plants with pollinators (P), the network connectance ($C_p = L_p / AP$, where L_p is the number of pollination links), and additional shape parameters *pcomp* and *pnest*. We used P = 3, 4, …, to 19 plants with pollinators, corresponding to 15 to 95% of the original 20 plants in the niche-model food webs (main text Figs. 2a, 2c). For simplicity, we fixed the ratio of animal-to-plant species to the value derived from the aggregated empirical networks (A/P = 2), yielding plant-pollinator network diversities of $S_p = A + P$ = 9, 12, …, to 57. For each S_p, we subdivided the range of connectances between $C_{p_{\min}}$ and $C_{p_{\max}}$ into 10 intervals, giving 11 values for C_p, inclusive of endpoints. Parameters *pcomp* and *pnest* are probabilities that determine rules by which plants and pollinators are linked together (see ref. [6], Supplementary Online Methods). Increasing *pcomp* towards 1 leads to more modular networks, i.e. networks where groups of species interact more among themselves than with species from other groups. Increasing *pnest* towards 1 leads to more nested networks, i.e. networks wherein specialist species tend to interact with partners that are a subset of the partners of more generalized species. Empirical mutualistic networks tend to be highly nested (see *Network properties*, below), but this property varies substantially between similar empirical networks and is strongly related to their diversity and connectance. We ran Thébault and Fontaine's [6] algorithm 10 times for each of the 11 C_p's corresponding to each S_p, with *pnest* varied from 0, 0.2, …, to 1 and *pcomp* fixed to 0 for a total of 10*11*17*5 = 9,350 networks. Only those networks in which each species had at least one link were retained. In particular, the confidence intervals for low S_p networks include values for C_p in which connectance is too low for all species to have at least one partner; these networks were excluded. From the remaining networks, we chose networks at each diversity level that most evenly sampled the empirical range of nestedness and connectance (Supplementary Fig. 1a-b). This resulted in approximately

14 plant-pollinator networks at each of the 17 initial diversity levels (S_P = 9, 12, ..., 57), for a total of 238 networks.

Network properties

Nestedness is a frequently observed, but controversial property of empirical mutualistic networks that has been hypothesized to endow them with both stability and function [5]. However, its effect appears to be dependent on both model assumptions [5,7–12] and its formal definition including its potential correlation to degree heterogeneity [13]. We therefore quantified both nestedness and degree heterogeneity of our simulated plant-pollinator networks (Supplementary Fig. 1b).

Specifically, we quantified nestedness as overlap and decreasing fill (NODF), standardized to the mean NODF of 100 iterations of the 'CE' null model in the ANINHADO software package [14,15]. This null model creates randomized matrices with the probability of a link between *i*, *j* set to the mean fraction of links realized in the *i*th row and *j*th column of our simulated interaction matrix. Standardized nestedness ranges from -1.0 to 1.1 in our simulated networks.

We defined 'degree heterogeneity' as the standard deviation of the degree distribution of all species (plants and pollinators), so that a more positive value indicates a more heterogeneous network. Degree heterogeneity ranges from 0.67 to 5.21 in our simulated networks and is significantly correlated with nestedness (R = 0.829, N = 238, $P < 0.0001$), as expected. Modularity is also a potentially stabilizing property of ecological networks though it tends to be inversely related to nestedness and is more often observed in herbivory networks than in mutualistic networks [6]. We quantified bipartite modularity in our plant-pollinator networks using the Newman's leading eigenvector method [16] (Q) standardized by the mean of 100 iterations of the 'Fixed' null model in the BiMat software package [17]. This null model swaps rows and columns of our simulated interaction matrix to create randomized matrices with the exact same row and column sums. Standardized modularity ranges from -0.34 to 0.64 in our simulated networks and is negatively correlated with nestedness (R = 0.451, N = 238, P < 0.0001).

Uncertainty & sensitivity analyses.

We explored the behavior of our multiplex model and assessed its robustness by performing uncertainty and sensitivity analyses on its free parameters (Supplementary Table 4). For these analyses, we simulated the N = 24,276 networks from each of the Rewards Only (RO) Food Web (FW), RO multiplex, and Rewards Plus (RP) multiplex treatments for 2000 timesteps, which was approximately dynamical steady-state (Fig. 3, Supplementary Figs. 3-4).

For the uncertainty analyses (Supplementary Table 1), we varied only one parameter varied at a time with all other parameters fixed to the main text values recorded in Supplementary Table 4, but with two exceptions: the half-saturation density fixed to the same value for all consumers (B_0 = 60), and the rewards productivity rate fixed to High (β = 1), unless otherwise noted. The qualitative effect of variation in each parameter is summarized in Supplementary Table 3 and quantitative results are given in Supplementary Table 1. A particularly important result, the effect of rewards productivity rate (β) on species persistence, is shown in Supplementary Fig. 2. For the sensitivity analyses (Supplementary Table 2), we varied all parameters simultaneously using a Latin Hypercube Sampling (LHS) design. Following Thébault and Fontaine [6], we varied the parameters between ¼ to 4 times the value used in the main text (Supplementary Table 4), but with β = 0.6 and B_0 = 60 for all consumers.

As reported in other work [19,20], the form of the functional response for feeding (main text Eqn. 3) as controlled by the Hill coefficient (h) and the half-saturation density (B_{0ij}) can modify the diversity-persistence relationship in complex food webs (Supplementary Tables 1-3). Otherwise, our results are most strongly mediated by food availability for pollinators (Supplementary Table 3), which is directly affected by rewards productivity (β) and rewards self-limitation (s) rates and indirectly affected by the cost of rewards production to vegetative total growth (κ) and community-wide carrying capacity of plant vegetation (K). In general, increasing rewards availability *directly* (increasing β and/or decreasing s) increases species persistence (e.g., Supplementary Fig. 2), pollinator biomass, and biomass of omnivores and carnivores so that they exceed that of FWs and furthermore allows for a positive diversity-persistence relationship. *Indirectly* increasing rewards availability (decreasing κ and/or increasing K) increases persistence, but changes the shape of how biomass accumulates in ecosystems with increasing initial diversity so that the diversity-persistence relationship shifts to negative; this may be an example of the paradox of enrichment. Regardless of the choice of parameters, multiplex networks tend to exhibit higher total biomass and productivity than the RO FW treatment, and increasing rewards availability tends to correspond to an increasingly positive relationship between initial diversity (S) and species persistence.

We additionally explored the effect of basic network structural properties on persistence in all N = 145,656 simulations in our six main text treatments. Specifically, we applied a Classification and Regression Tree (CART) analysis with five-folded cross-validation to predict species persistence at the end of simulations [18] given the following metrics of initial network structure: network treatment (RO or RP), rewards productivity level (β = 0 [FW], 0.2, 1), initial diversity (S), connectance of the network (C), connectance of the niche-model food web (C_f), connectance of the plant-pollinator network (C_p), nestedness of the plant-pollinator network (NODFst and degree heterogeneity), and modularity of the plant-pollinator network (Q and Qst). All inputs are continuous variables except for network treatment and rewards productivity level, which are categorical. See Supplementary Table 4 for definitions of structural metrics.

Rewards productivity accounted for 73% of the explained variance (five-folded R^2 = 0.48, N = 145,656), while network connectance (C) and initial diversity (S) accounted for ~9% each, and other network metrics including nestedness and modularity accounted for < 2% each. Subdividing our networks by initial diversity (S) yields similar results. For example, when S = 64, rewards productivity accounted for 73% while C_f and C each account for ~11% of the explained variance in persistence (five-folded R^2 = 0.37, N = 8568). When S = 80, rewards productivity accounted for 85% and C accounts for 10% of the explained variance in persistence (five-folded R^2 = 0.61, N = 9180). In both cases, metrics of nestedness and modularity explain < 4% of explained variance in observed species persistence. This underscores the importance of rewards productivity (β) for species persistence in our simulations.

SUPPLEMENTARY DISCUSSION

In general (Supplementary Tables 1-3), multiplex networks above a certain threshold of food availability for pollinators displayed increased persistence and biomass abundance compared to the RO FW treatment, indicating that this result is robust to variation in parameter values, though the qualitative relationship between initial diversity (S) and species persistence may vary. The effects of food availability for pollinators are most obvious in the Rewards Only (RO) multiplex treatment, where increasing food availability increases pollinator abundance and

subsequently the abundance and persistence of omnivores and carnivores. On the other hand, in the Rewards Plus (RP) multiplex treatment, increasing food availability increases pollinator abundance, which increases the abundance and persistence of omnivores and carnivores but additionally leads pollinators to exert increased direct competition and predation pressure on lower trophic-level consumers (herbivores and omnivores), reducing species persistence and abundance in the ecosystem overall. However, the RP multiplex treatment has increased persistence and abundance at low pollinator food availability than analogous RO networks. See Supplementary Fig. 6 for an example of this pattern driven by rewards productivity (β).

This hypothesis is also coherent with the results of our feedback controls. Overall, ecosystem diversity, persistence, biomass, and productivity in the feedback controls equilibrate to similar values as in the multiplex simulations (Supplementary Figs. 6-8). The only overall difference was increased vegetative biomass of plants with pollinators and decreased biomass of plants without pollinators in the feedback controls (Supplementary Fig. 8d). Though plants with pollinators in multiplex treatments can potentially achieve higher growth rates than plants without pollinators in the presence of sufficient reproductive services, plants with pollinators in feedback controls increase in biomass because they are not subjected to dynamic effects of mutualism such as variable vegetative growth rate and costly rewards production. All controls except High RP additionally displayed decreased omnivore persistence and biomass, an effect that was most prominent in the Low rewards controls (Supplementary Fig. 8b, 8d). We also observed decreased herbivore and increased pollinator persistence in the RO controls and decreased added-omnivore/pollinator persistence in the Low RP control (Supplementary Fig. 8b). These changes appear related to transient oscillations in species' biomass. The period and amplitude of pollinators' and plants' oscillations in the controls are less synchronized and decrease (e.g. Supplementary Fig. 9) presumably due to steadier rewards biomass in the absence of its dynamic coupling to vegetation. However, omnivores, added-omnivores/pollinators in RP controls, and herbivores have increased oscillations with increased synchrony. We hypothesize that transient oscillations between mutualists may stabilize multiplex networks by increasing compensatory dynamics in consumers' resources which is especially important for species that integrate over many of the resources within an ecosystem such as omnivores. These guild-level differences were notable in the RO and Low RP treatments but tiny in the High RP treatment.

An interesting possibility suggested by these results is that the increased trophic links in RP networks may dampen the effects of mutualistic feedbacks especially when rewards are sufficiently available, i.e. at High rewards productivity. This is also suggested by the smaller ranges in outcomes between High and Low RP treatments compared to the larger range in High and Low RO treatments (Fig. 4, Supplementary Fig. 2).

Supplementary Table 1: Parameters and quantitative results of uncertainty analyses. Species persistence resulting from varying each unconstrained parameter in our simulations at a time. Persistence reported as Average ± Standard Deviation (N = 24,276) and Slope, R^2 of the Initial Diversity (S) versus Persistence relationship for three network treatments: Rewards Only (RO) Multiplex and Food Webs (FW) and Rewards Plus (RP) Multiplex. Parameters were varied from the main text values (see Table S4), but with simulation length reduced to 2000 timesteps, and, unless otherwise noted, β_i fixed to 1 (corresponding to the High rewards productivity treatments) and B_{0ij} fixed to 60. For reference, gray highlighted rows show results using the main text parameter combinations but with B_{0ij} fixed to 60. Bolded entries show positive diversity-persistence relationships.

	Rewards Only (RO)				Rewards Plus (RP)	
Parameter	Food Web (FW)		Multiplex		Multiplex	
	Persistence	S vs. Persist	Persistence	S vs. Persist	Persistence	S vs. Persist
**β**						
0.1			0.40 ± 0.11	−0.0071, 0.37	0.45 ± 0.10	−0.0062, 0.42
0.2	0.53 ± 0.14	−0.0015, 0.01	0.41 ± 0.12	−0.0065, 0.26	0.43 ± 0.12	−0.0070, 0.31
0.4			0.56 ± 0.13	−0.0018, 0.02	0.52 ± 0.10	−0.0046, 0.19
0.6			0.62 ± 0.17	**+0.0006, 0.00**	0.55 ± 0.12	−0.0023, 0.03
0.8			0.66 ± 0.13	**+0.0007, 0.00**	0.54 ± 0.11	−0.0044, 0.15
1.0	0.53 ± 0.14	−0.0015, 0.01	0.72 ± 0.17	**+0.0045, 0.07**	0.62 ± 0.12	−0.0007, 0.00
1.4			0.75 ± 0.16	**+0.0058, 0.13**	0.65 ± 0.12	**+0.0003, 0.00**
1.8			0.76 ± 0.15	**+0.0065, 0.17**	0.66 ± 0.12	**+0.0011, 0.01**
**s**						
0.1			0.67 ± 0.13	**+0.0010, 0.01**	0.55 ± 0.11	−0.0046, 0.15
0.2			0.75 ± 0.16	**+0.0050, 0.10**	0.64 ± 0.12	**+0.0000, 0.00**
0.4	0.53 ± 0.14	−0.0015, 0.01	0.72 ± 0.17	**+0.0045, 0.07**	0.62 ± 0.12	−0.0007, 0.00
0.8			0.63 ± 0.17	**+0.0024, 0.02**	0.56 ± 0.13	−0.0003, 0.00
1.6			0.59 ± 0.14	**+0.0013, 0.01**	0.52 ± 0.11	−0.0050, 0.19
**κ**						
0.01			0.75 ± 0.12	−0.0026, 0.05	0.55 ± 0.16	−0.0048, 0.16
0.05			0.72 ± 0.13	−0.0006, 0.00	0.55 ± 0.12	−0.0046, 0.15
0.10	0.53 ± 0.14	−0.0015, 0.01	0.72 ± 0.17	**+0.0045, 0.07**	0.62 ± 0.12	−0.0007, 0.00
0.25			0.64 ± 0.14	**+0.0032, 0.05**	0.56 ± 0.12	−0.0001, 0.00
0.50			0.56 ± 0.12	**+0.0007, 0.00**	0.52 ± 0.11	−0.0016, 0.02
1.00			0.51 ± 0.09	−0.0023, 0.06	0.49 ± 0.11	−0.0043, 0.15
**B_0i,j**						
20	0.77 ± 0.14	−0.0078, 0.28	0.79 ± 0.13	−0.0043, 0.11	0.73 ± 0.15	−0.0064, 0.17
40	0.69 ± 0.14	−0.0081, 0.34	0.85 ± 0.10	−0.0018, 0.03	0.72 ± 0.13	−0.0041, 0.10
60	0.53 ± 0.14	−0.0015, 0.01	0.72 ± 0.17	**+0.0045, 0.07**	0.62 ± 0.12	−0.0007, 0.00
80	0.33 ± 0.10	**+0.0018, 0.03**	0.46 ± 0.19	**+0.0111, 0.34**	0.45 ± 0.14	**+0.0060, 0.16**

100	0.29 ± 0.05	–0.0028, 0.26	0.37 ± 0.14	**+0.0067, 0.22**	0.37 ± 0.12	**+0.0046, 0.15**
**B_0i,rewards**						
20			0.75 ± 0.15	**+0.0051, 0.10**	0.65 ± 0.12	**+0.0003, 0.00**
40			0.74 ± 0.16	**+0.0048, 0.09**	0.63 ± 0.12	–0.0004, 0.00
60	0.53 ± 0.14	–0.0015, 0.01	0.72 ± 0.17	**+0.0045, 0.07**	0.62 ± 0.12	–0.0007, 0.00
80			0.70 ± 0.17	**+0.0040, 0.05**	0.60 ± 0.13	–0.0009, 0.01
100			0.67 ± 0.18	**+0.0034, 0.04**	0.58 ± 0.13	–0.0010, 0.01
**generalist model**						
**ω consumers,j**						
weak	0.53 ± 0.14	–0.0015, 0.01	0.72 ± 0.17	**+0.0045, 0.07**	0.62 ± 0.12	–0.0007, 0.00
strong			0.61 ± 0.15	–0.0044, 0.08	0.55 ± 0.14	–0.0063, 0.20
**ω pollinators,j**						
weak	0.53 ± 0.14	–0.0015, 0.01	0.72 ± 0.17	**+0.0045, 0.07**	0.62 ± 0.12	–0.0007, 0.00
strong			0.67 ± 0.13	**+0.0014, 0.01**	0.55 ± 0.11	–0.0043, 0.14
**h**						
1.0	0.24 ± 0.08	–0.0070, 0.73	0.20 ± 0.08	–0.0057, 0.45	0.17 ± 0.08	–0.0050, 0.41
1.2	0.56 ± 0.12	–0.0063, 0.25	0.71 ± 0.15	–0.0040, 0.07	0.56 ± 0.14	–0.0074, 0.25
1.5	0.53 ± 0.14	–0.0015, 0.01	0.72 ± 0.17	**+0.0045, 0.07**	0.62 ± 0.12	–0.0007, 0.00
2.0	0.34 ± 0.11	**+0.0029, 0.07**	0.52 ± 0.19	**+0.0124, 0.39**	0.50 ± 0.14	**+0.0056, 0.16**
**K**						
120	0.28 ± 0.04	–0.0040, 0.98	0.29 ± 0.04	–00028, 0.38	0.31 ± 0.07	–00009, 0.02
240	0.28 ± 0.04	–0.0040, 0.98	0.33 ± 0.10	**+0.0026, 0.08**	0.40 ± 0.11	**+0.0015, 0.02**
360	0.33 ± 0.10	**+0.0018, 0.03**	0.43 ± 0.17	**+0.0096, 0.29**	0.43 ± 0.14	**+0.0053, 0.14**
480	0.53 ± 0.14	–0.0015, 0.01	0.72 ± 0.17	**+0.0045, 0.07**	0.62 ± 0.12	–0.0007, 0.00
540	0.61 ± 0.12	–0.0062, 0.26	0.74 ± 0.11	–0.0020, 0.03	0.56 ± 0.12	–0.0047, 0.15
600	0.65 ± 0.12	–0.0073, 0.33	0.85 ± 0.11	–0.0005, 0.00	0.69 ± 0.12	–0.0028, 0.05
720	0.69 ± 0.14	–0.0081, 0.34	0.79 ± 0.11	–0.0038, 0.12	0.57 ± 0.12	–0.0051, 0.17
840	0.71 ± 0.14	–0.0083, 0.33	0.89 ± 0.10	–0.0012, 0.01	0.75 ± 0.12	–0.0036, 0.08
960	0.73 ± 0.14	–0.0082, 0.32	0.89 ± 0.10	–0.0013, 0.02	0.77 ± 0.12	–0.0036, 0.09
1320	0.76 ± 0.14	–0.0079, 0.29	0.89 ± 0.10	–0.0016, 0.02	0.79 ± 0.12	–0.0037, 0.09

Supplementary Table 2: Parameters and quantitative results of sensitivity analyses. Parameters combinations (rows) were constructed using a Latin Hypercube Sampling (LHS) procedure. For each parameter, 18 samples were drawn randomly from a uniform distribution of $[1/4x, 4x]$ where x is the value used in the main text simulations (Table S4, but with β = 0.6, B_{0ij} = 60). Only K and B_0 (gray-shaded columns) are relevant parameters for the Food Web (FW) treatments. For all simulations, h was fixed to 1.5 and r was fixed to 0.8 for plants without pollinators or 1.0 for plants with pollinators. Bolded entries show positive diversity-persistence relationships. Persistence is reported as Average (Avg) and Standard Deviation (SD) of N = 24,276 simulations and Slope, R^2 of the Initial Diversity (S) versus Persistence relationship for three network treatments: Rewards Only (RO) Multiplex and Food Webs (FW) and Rewards Plus (RP) Multiplex. Rows are ordered by increasing K in the parameter combinations.

Parameter Combinations						Rewards Only (RO)								Rewards Plus (RP)			
						Food Web (FW)				Multiplex				Multiplex			
						Persistence		S vs. Persist		Persistence		S vs. Persist		Persistence		S vs. Persist	
K	B_0	β	s	κ	benefit coefficient	Avg	SD	Slope	R^2	Avg	SD	Slope	R^2	Avg	SD	Slope	R^2
205.4	76.72	2.063	1.262	0.350	0.130	0.28	0.04	–0.0040	0.98	0.28	0.04	–0.0038	0.92	0.28	0.04	–0.0038	0.91
308.9	204.93	3.037	0.682	0.196	0.065	0.28	0.04	–0.0040	0.98	0.29	0.05	–0.0034	0.53	0.28	0.04	–0.0037	0.86
416.4	169.74	3.023	0.929	0.371	0.050	0.28	0.04	–0.0040	0.98	0.30	0.06	–0.0019	0.11	0.29	0.04	–0.0030	0.53
433.7	230.60	1.692	1.529	0.394	0.032	0.28	0.04	–0.0040	0.98	0.28	0.04	–0.0040	0.98	0.28	0.04	–0.0040	0.98
611.6	130.54	1.435	0.250	0.034	0.043	0.29	0.05	–0.0032	0.42	0.44	0.22	**+0.0120**	0.29	0.45	0.17	**+0.0076**	0.19
697.5	85.67	2.564	1.362	0.155	0.171	0.54	0.14	–0.0025	0.03	0.69	0.15	**+0.0056**	0.13	0.60	0.13	**+0.0006**	0.00
720.0	221.24	0.499	0.144	0.336	0.121	0.28	0.04	–0.0040	0.98	0.31	0.06	–0.0006	0.01	0.30	0.04	–0.0020	0.24
891.6	193.12	1.834	1.004	0.272	0.079	0.29	0.05	–0.0035	0.56	0.33	0.09	**+0.0024**	0.06	0.32	0.07	**+0.0008**	0.01
989.8	100.18	0.991	1.513	0.180	0.194	0.65	0.12	–0.0072	0.32	0.63	0.12	–0.0043	0.13	0.55	0.14	–0.0076	0.27
1084.5	144.91	2.726	1.320	0.049	0.016	0.46	0.14	**+0.0016**	0.01	0.77	0.20	**+0.0086**	0.17	0.65	0.14	**+0.0011**	0.01
1201.1	110.54	0.795	0.462	0.308	0.108	0.67	0.13	–0.0078	0.34	0.78	0.12	–0.0015	0.02	0.66	0.13	–0.0043	0.10
1302.1	120.84	0.221	1.118	0.136	0.159	0.67	0.13	–0.0078	0.33	0.49	0.14	–0.0113	0.65	0.50	0.17	–0.0140	0.64
1354.2	60.53	1.317	0.819	0.217	0.104	0.76	0.14	–0.0078	0.28	0.88	0.10	–0.0019	0.03	0.77	0.13	–0.0046	0.13
1463.0	33.20	0.613	1.019	0.117	0.064	0.79	0.15	–0.0068	0.21	0.74	0.14	–0.0053	0.13	0.71	0.16	–0.0081	0.24
1521.2	40.75	2.437	0.395	0.276	0.143	0.79	0.14	–0.0071	0.23	0.84	0.13	–0.0020	0.02	0.78	0.13	–0.0046	0.11

1670.2	21.72	2.306	0.290	0.077	0.091	0.79	0.15	−0.0061	0.16	0.85	0.17	−0.0029	0.03	0.78	0.15	−0.0037	0.06
1752.2	153.91	1.076	0.565	0.088	0.157	0.68	0.13	−0.0079	0.33	0.87	0.10	−0.0011	0.01	0.71	0.13	−0.0035	0.07
1907.8	182.42	1.980	0.691	0.246	0.180	0.66	0.13	−0.0076	0.33	0.78	0.12	**+0.0013**	0.01	0.67	0.13	−0.0022	0.03

Supplementary Table 3: Qualitative effects of variation in parameter values on persistence, abundance, and productivity.

Symbol	Main Text Value [Min., Max.]	Definition	Effect on Persistence	Observations & Hypothesized Mechanism
β_i	Low = 0.2, High = 1; None corresponds to FWs [0.1, 1.8]	Production rate of plant with pollinator *i*'s floral rewards	↑	↑ persistence & biomass of pollinators and their predators by ↑ food availability for pollinators; shifts multiplex networks to + diversity-persistence relationship
s_i	0.4 [0.1, 1.6]	Self-limitation rate of rewards production for plant with pollinator species *i*	↓	↓ food availability for pollinators by slowing the recovery rate of depleted rewards, ↓ persistence & biomass of pollinators and their predators by ↑ competition among pollinators for resources
κ_i	0.1 [0.01, 1]	Cost of producing floral rewards biomass for plant with pollinator species *i* in terms of vegetative biomass	~↓	Stronger coupling of rewards production to vegetative growth rate; nonmonotonic effect on rewards but generally ↓ biomass of pollinators & plants with pollinators' vegetation, & ↓ pollinator persistence by ↓ their food availability
B_{0ij} when *j* is species biomass	60 [20, 100]	Half-saturation density; density of *j* at which *i* consumes at half its maximum feeding rate on *j*	↓	Slows consumption by *i* on rare resources, ↓ biomass & persistence of consumers at low diversity; shifts networks to + diversity-persistence relationship
B_{0ij} when *j* is rewards	30 [20, 100]	Same as above	↓	Slows consumption by pollinator *i* on rare rewards, ↑ rewards biomass, ↓ biomass & diversity of pollinators, especially at low diversity
h	1.5 [1, 2]	Hill coefficient, determines the shape of F_{ij}	↑	Decelerates consumption on rare resources (Martinez *et al.* 2006); nonmonotonic effect on biomass, shifts networks to + diversity-persistence relationship
K	480 [120, 1320]	Plant community-wide carrying capacity	↑	↑ basal food availability (vegetation); shifts networks to + then – diversity-persistence relationship

Qualitative effects of each parameter as recorded from uncertainty analyses, where the focal parameter was increased from a Minimum to Maximum value (in brackets under the Main Text Value) while all other parameters were fixed. Patterns in persistence, biomass, production, and consumption when the parameter is increased are recorded as observations; hypothesized mechanisms leading to these patterns are also provided. If increasing the parameter modifies the qualitative relationship between initial diversity (*S*) and persistence, it is also recorded. Pollination parameters (β, s, κ, B_{0ij} when *j* is rewards) are only relevant for multiplex networks. Remaining (h, K, and B_{0ij} when *j* is not rewards) hold for all treatments.

Supplementary Table 4: Summary of symbols, definitions, and values used in the main text.

Symbol	Definition	Main Text Value	References
**Network architecture.**			
S_f	Niche-model food web diversity (num. species)	50 with exactly 20 plants and 5 herbivores	Fig. 1a
L_f	Num. directed feeding links	$244 < L_f < 256$	
C_f	Niche-model food web directed connectance	$L_f / S_f^2 = 0.1$	Martinez 1991
P	Num. plants with pollinator species	3, 4, …, 19	
A	Num. animal-pollinator species	$2P = 6$, 8, …, 38	
S_p	Plant-pollinator network diversity	$P + A = 9$, 12, …, 57	Fig. 1b
L_p	Num. directed pollination links	Range for each S_p set by empirical networks	
C_p	Plant-pollinator network directed connectance	L_p / PA = range for each S_p set by empirical networks	
S	Diversity of network treatments	$S_f + 2/3(S_p) = 56$, 58, …, 88	Fig. 1c
C	Directed connectance of network treatments	Range for each S set by plant-pollinator network and inherited niche model links	Fig. 1d-e
**Network dynamics and parameterization.**			
B_i	Biomass of species *i*	Evaluated numerically from the system of differential equations	Eqns. 1, 4, 5
R_i	Floral rewards biomass of plant with pollinator *i*	Same as B_i	Eqn. 6
K	Plant community-wide carrying capacity	480	
$C_{ij}(B_j)$ or $C_{ij}(R_j)$	Consumption rate of species *i* eating species *j* or *j*'s floral rewards, respectively		Eqn. 2
F_{ij}	Functional response for *i* eating *j*, describing the realized fraction of *j*'s biomass that is consumed as a function of *i*'s preference for *j* and *j*'s prevalence		Eqn. 3
ω_{ij}	Preference of *i* for eating *j*	1/(*i*'s diet size), a.k.a. 'the weak generalist model'	Williams 2008
h	Hill coefficient, determines the shape of F_{ij}	1.5	Real 1977, Martinez *et al.* 2006
B_{0ij}	Half-saturation density; density of *j* at which *i* consumes at half its maximum feeding rate on *j*	60 when *j* is species biomass, 30 when *j* is rewards biomass	Boit *et al.* 2012
$P(R_i)$	Function describing plant with pollinator *i*'s accrual of *reproductive services* due to pollination	Of the form: *reproductive services* / (0.05 + *reproductive services*), where the shape parameter set to 0.05 is also called the *benefit coefficient*	Eqn. 7
β_i	Production rate of plant with pollinator *i*'s floral rewards	Rewards productivity treatments: Low = 0.2, High = 1	
s_i	Self-limitation rate of rewards production for plant with pollinator *i*	0.4	

κ_i	Cost of producing floral rewards biomass for plant with pollinator *i* in terms of its vegetative biomass production	0.1	
**Allometric parameterization.**			
$swTL_i$	Short-weighted trophic level of species *i*	*i* is a plant: 1, herbivore: 2, omnivore or carnivore: >2	Williams & Martinez 2004
x_i	Mass-specific metabolic rate of species *i*	*i* is a plant: 0, *i* is a consumer: $0.314m_i^{-0.25}$	Brose *et al. 2006,* Martinez *et al.* 2012
m_i	Body mass of species *i*	$Z^{swTL_i - 1}$ with *Z* sampled from lognormal distribution with mean = 10, std. dev. = 100	Martinez *et al.* 2012
r_i	Max. mass-specific growth rate of plant species *i*	Plants w/o pollinators: 0.8, plants w/ pollinators: 1	Martinez *et al.* 2012
y_{ij}	Max. metabolic-specific consumption rate of *i* eating *j*	10	Martinez *et al.* 2012
e_{ij}	Assimilation efficiency of *i* eating *j*	*j* is a plant w/o pollinators or vegetative biomass: 0.66, *j* is an animal: 0.85, *j* is floral rewards: 1	Martinez *et al.* 2012
**Simulation settings.**			
Initial Biomass	Biomass of B_i or R_i at the beginning of the simulation	10	
Simulation Length	Num. timesteps in a simulation	5000	
Solver	Method used to numerically evaluate ('simulate') differential equations specifying species' dynamics	Multiplex: ode15s, Food Web (FW): ode45 used with RelTol = 1^{-7}, AbsTol = 1^{-9}	MATLAB 2018b
Extinction Threshold	Biomass under which a species is considered extinct and its biomass is set to 0 for the remainder of the simulation	10^{-6}	

Supplementary Figure 3: Effects of increasing mutualism on plant persistence.

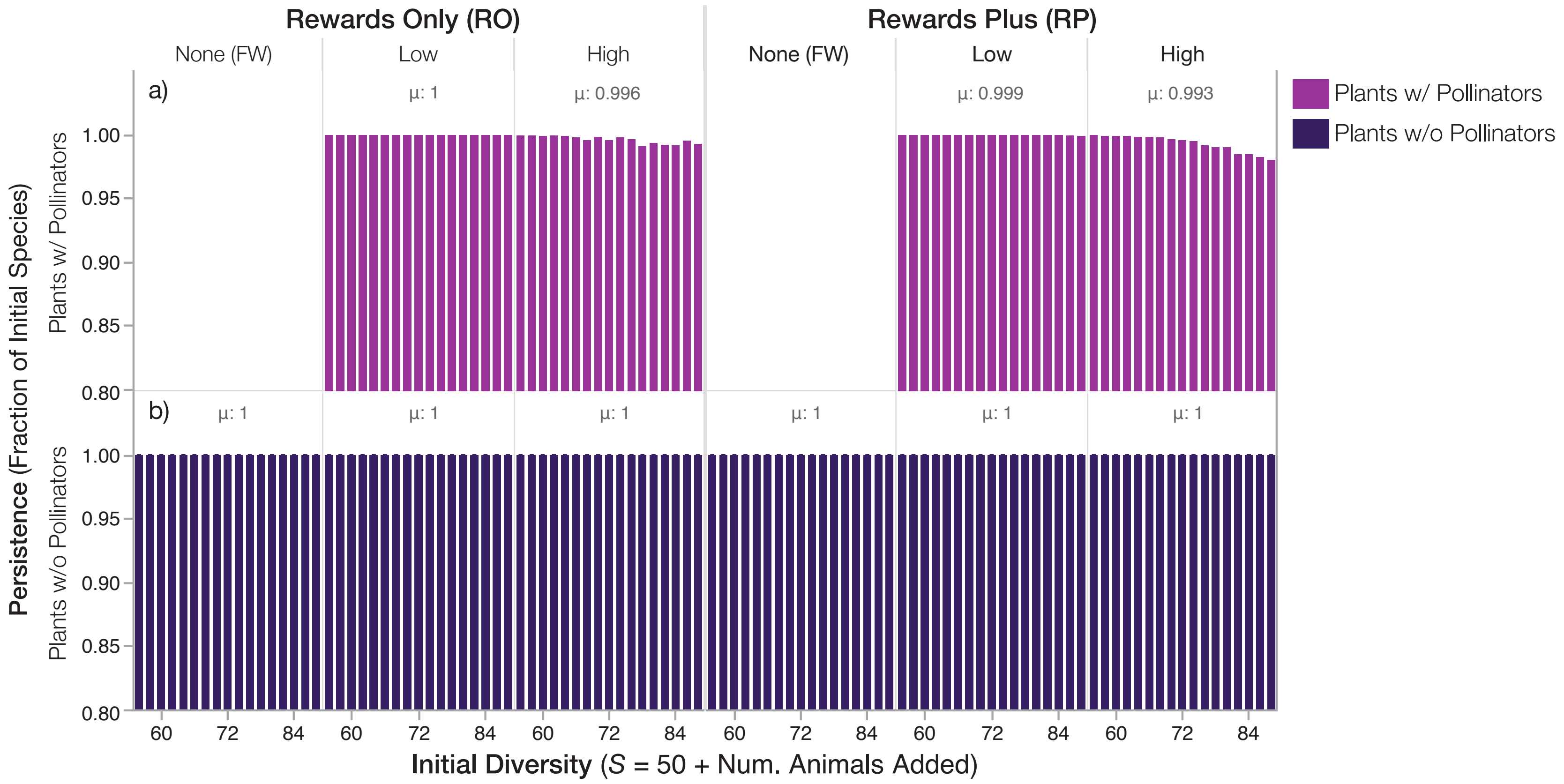

Guild persistence of a) plants with pollinators (purple) and b) plants without pollinators (dark purple), following the formatting of main text Fig. 5. Persistence is that fraction of initial species that persist (i.e. avoid extinction) to the end of simulations. Plants without pollinators always persist. Average persistence of plants with pollinators decreases with increasing diversity and mutualism, but overall extinctions are rare.

Supplementary Figure 4: Robustness of diversity and persistence to simulation length.

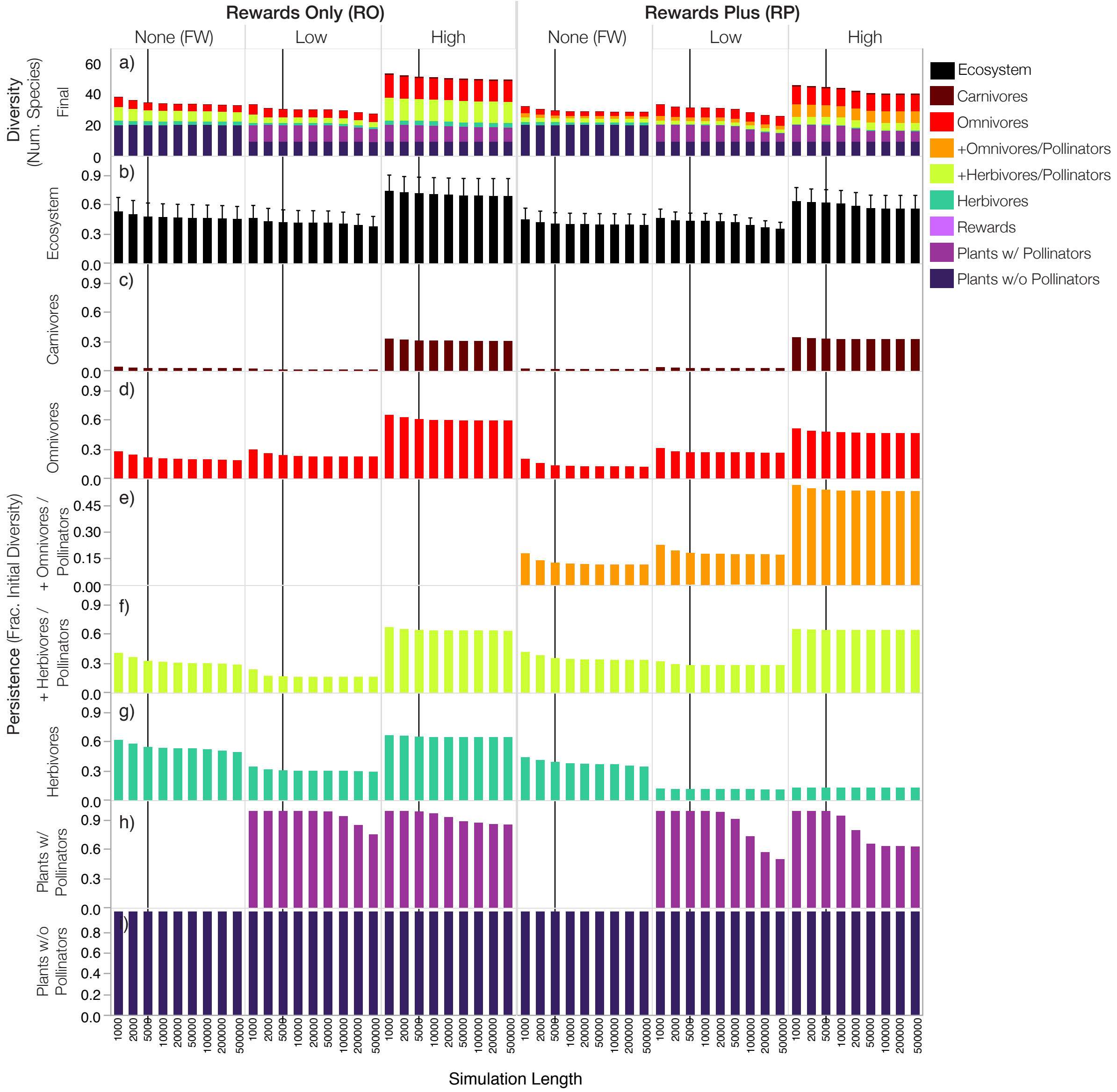

Persistence and diversity of a subset of our simulations evaluated at different simulation lengths (x-axis). The simulations are of 90 randomly sampled $S = 72$ networks subjected to the six network treatments. Formatting follows main text Fig. 5 with the inclusion of plant guild persistence as in Supplementary Fig. 3. Black vertical lines indicate results at 5000 timesteps, the length of the main text simulations. Error bars are standard deviations.

Supplementary Figure 5: Robustness of abundance, function, and temporal stability to simulation length.

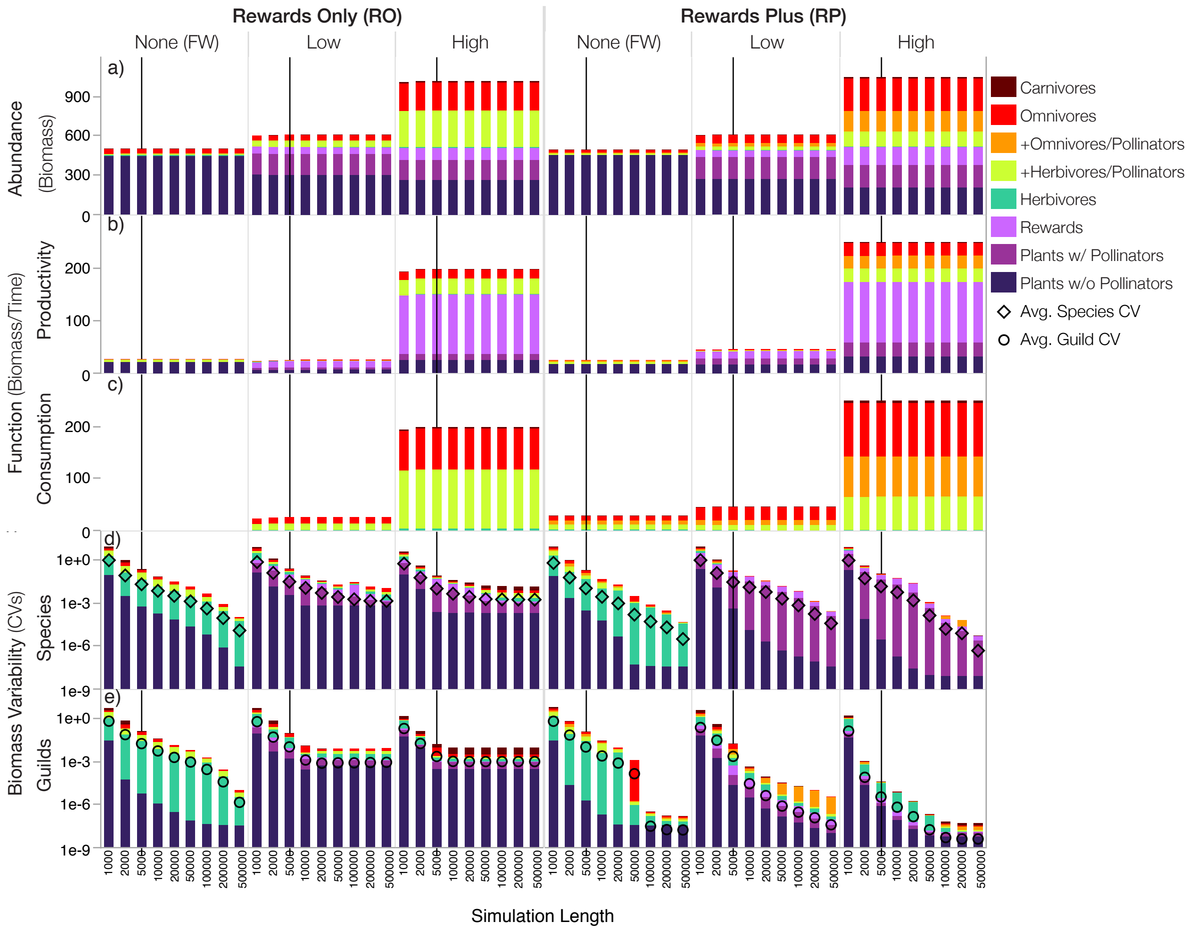

Persistence and diversity of a subset of our simulations evaluated at different simulation lengths (x-axis). The simulations are of 90 randomly sampled $S = 72$ networks subjected to the six network treatments. Formatting follows main text Fig. 6, with the inclusion of hollow circles and diamonds indicate average values corresponding to the μ reported for species and guild CVs in main text Fig. 4e-f. Black vertical lines indicate results at 5000 timesteps, the length of the main text simulations. Error bars are standard deviations.

Supplementary Figure 10: Connectance of network treatments.

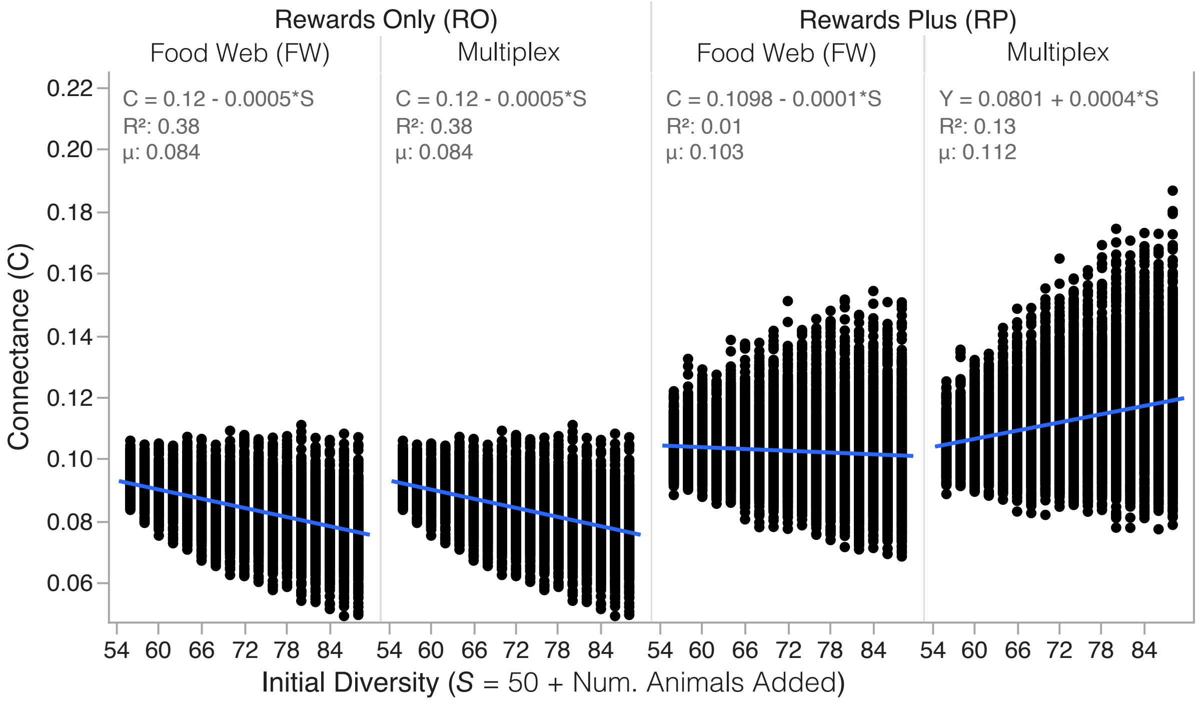

The relationship of connectance (*C*) with species diversity (*S*) in our network treatments as described by linear regression (blue lines, given by the equation and R^2) and mean (μ) over *S*. Connectance of the network treatments is defined as $C = L/S^2$, where *L* is the total number of links (both mutualistic and feeding) and *S* is the number of species in the network. Rewards Only (RO) treatments have identical connectance because every mutualistic link between pollinators and plants in the multiplex treatment is transformed into a feeding link by herbivores on plants in the Food Web (FW) treatment. Because the connectance of our simulated plant-pollinator networks decreases with increasing diversity (Supplementary Fig. 1a) and pollinators have no other resources, *C* decreases with increasing *S* in the Rewards Only treatments. In the Rewards Plus (RP) multiplex treatment, pollinators can be both a pollinator and an herbivore of a given plant. These two links are transformed into a single herbivory link in the RP Food Web (FW) treatment, leading to slightly reduced connectance in the FWs (~ 0.10) compared to the multiplex networks (~0.11). RP pollinators were allowed to be herbivorous or omnivorous (main text Fig. 2d) such that *C* remains approximately constant with increasing *S* in RP FWs. *N* = 24,276 network in each treatment.

Supplementary Figure 1: Properties of empirical and simulated plant-pollinator networks.

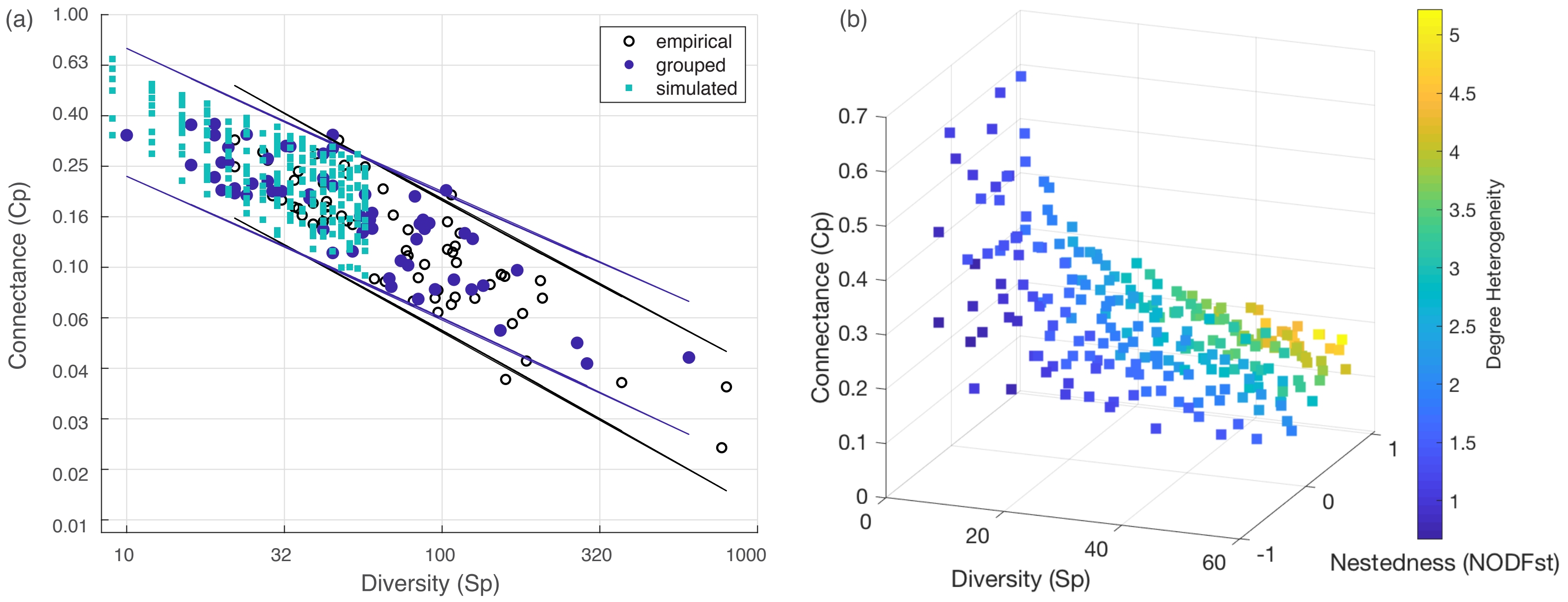

a) The relationship of connectance (C_p) with species diversity (S_p) in $N = 49$ empirical plant-pollinator networks before (black, open circles) and after (blue, filled circles) trophic grouping and the 95% confidence intervals around each (lines). Simulated plant-pollinator networks (squares) have properties bounded by the grouped empirical networks. Note the log-log axes. b) Properties of the 238 simulated plant-pollinator networks (squares). Connectance and diversity are highly correlated with nestedness and degree heterogeneity (colors).

Supplementary Figure 2: Persistence along a gradient of increasing floral rewards productivity.

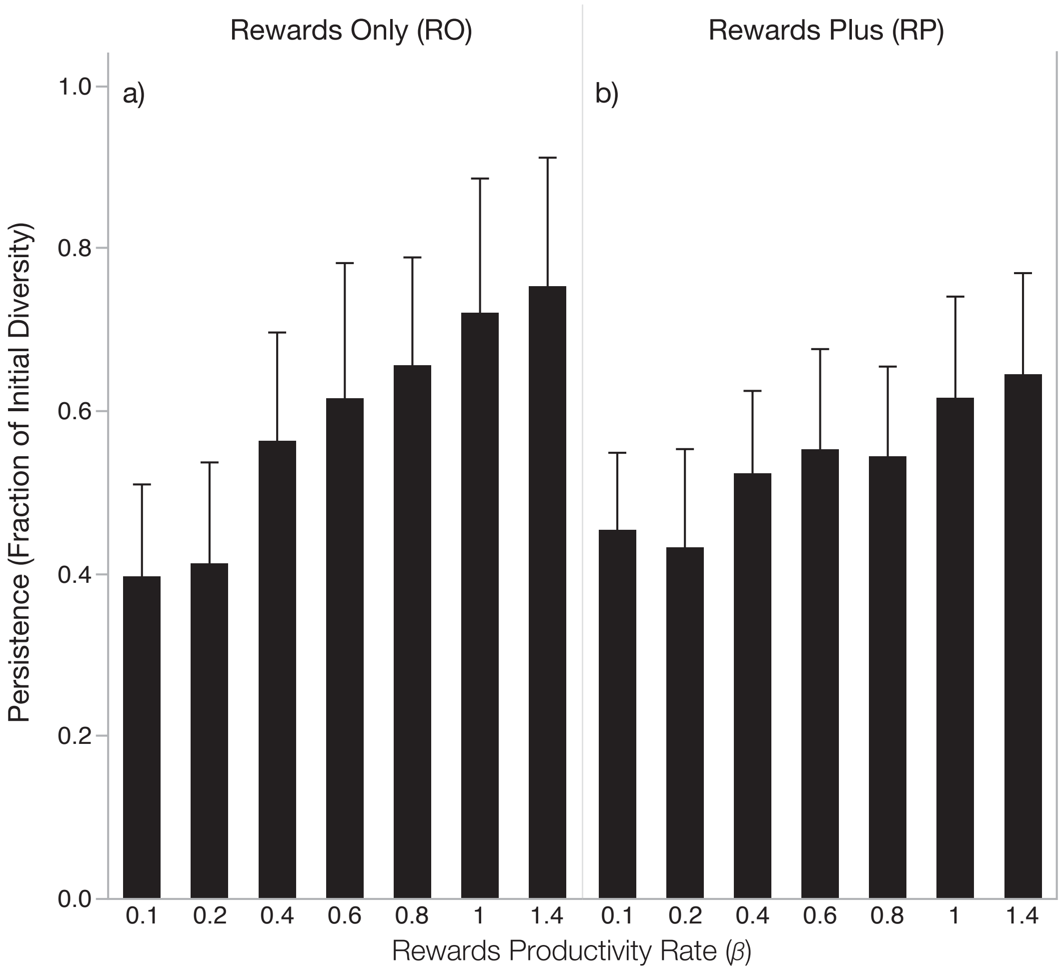

Mean (bars) and standard deviation (error bars) of species persistence at varying rewards productivity for the N = 24,276 networks each in the a) Rewards Only (RO) and b) Rewards Plus (RP) treatments. All others parameters were fixed. The fraction of species that survive to the end of the simulations (persistence) is bounded between 0 and 1. At very low rewards productivity (β), persistence is ~0.4, corresponding to nearly all animals going extinct. Persistence increases with increasing β, but tends to level off so that there is a smaller difference in average persistence between $\beta = 1$ and 1.4 than between $\beta = 0.8$ and 1 (notice the non-linear x-axis). Persistence in RO networks is more affected by β than in RP networks.

Supplementary Figure 6: Diversity and persistence in feedback controls.

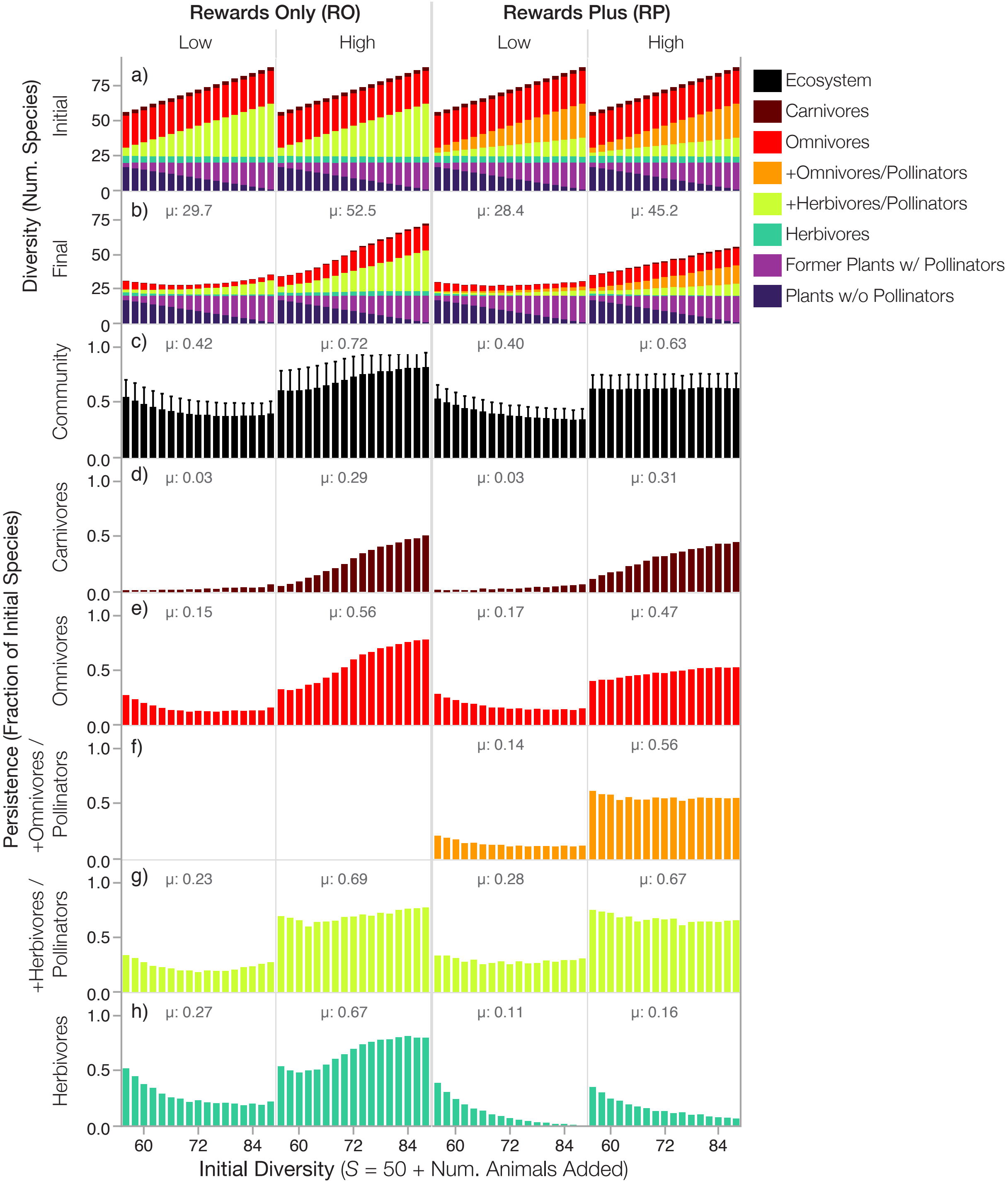

Full results for the feedback control treatments, in which food webs are initialized with rewards productivity from steady-state multiplex networks. Formatting follows main text Fig. 5 with purple indicating *former* plants with pollinators. μ's are means calculated corresponding to values presented in main text Fig. 4. Error bars show standard deviations.

Supplementary Figure 7: Abundance, function, and temporal stability in feedback controls.

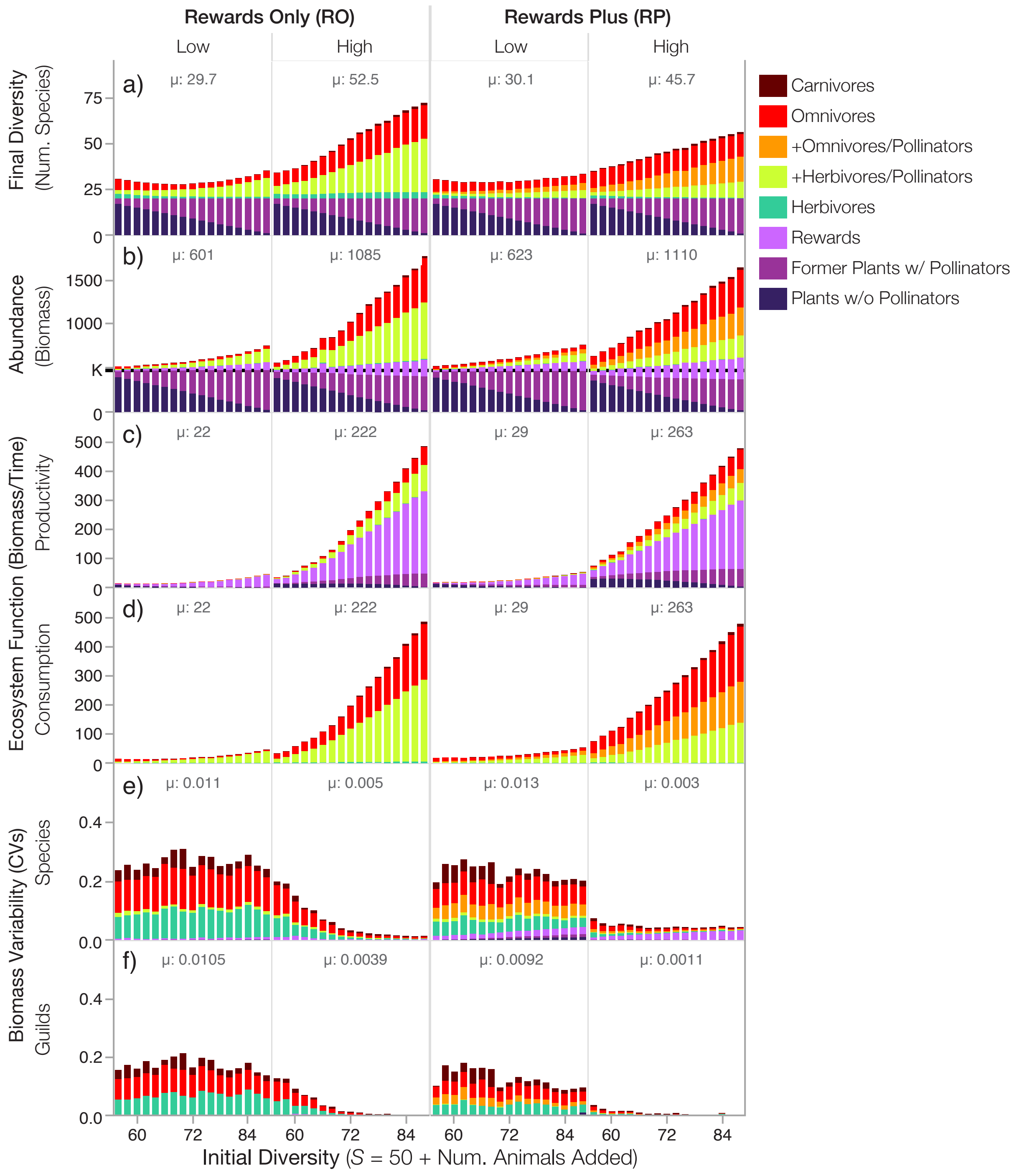

Full results for the feedback control treatments, in which food webs are initialized with rewards productivity from steady-state multiplex networks. Formatting follows main text Fig. 6 with purple indicating *former* plants with pollinators. μ's are means with calculated corresponding to values presented in main text Fig. 4. Error bars show standard deviations.

Supplementary Figure 8: Composition changes in feedback controls.

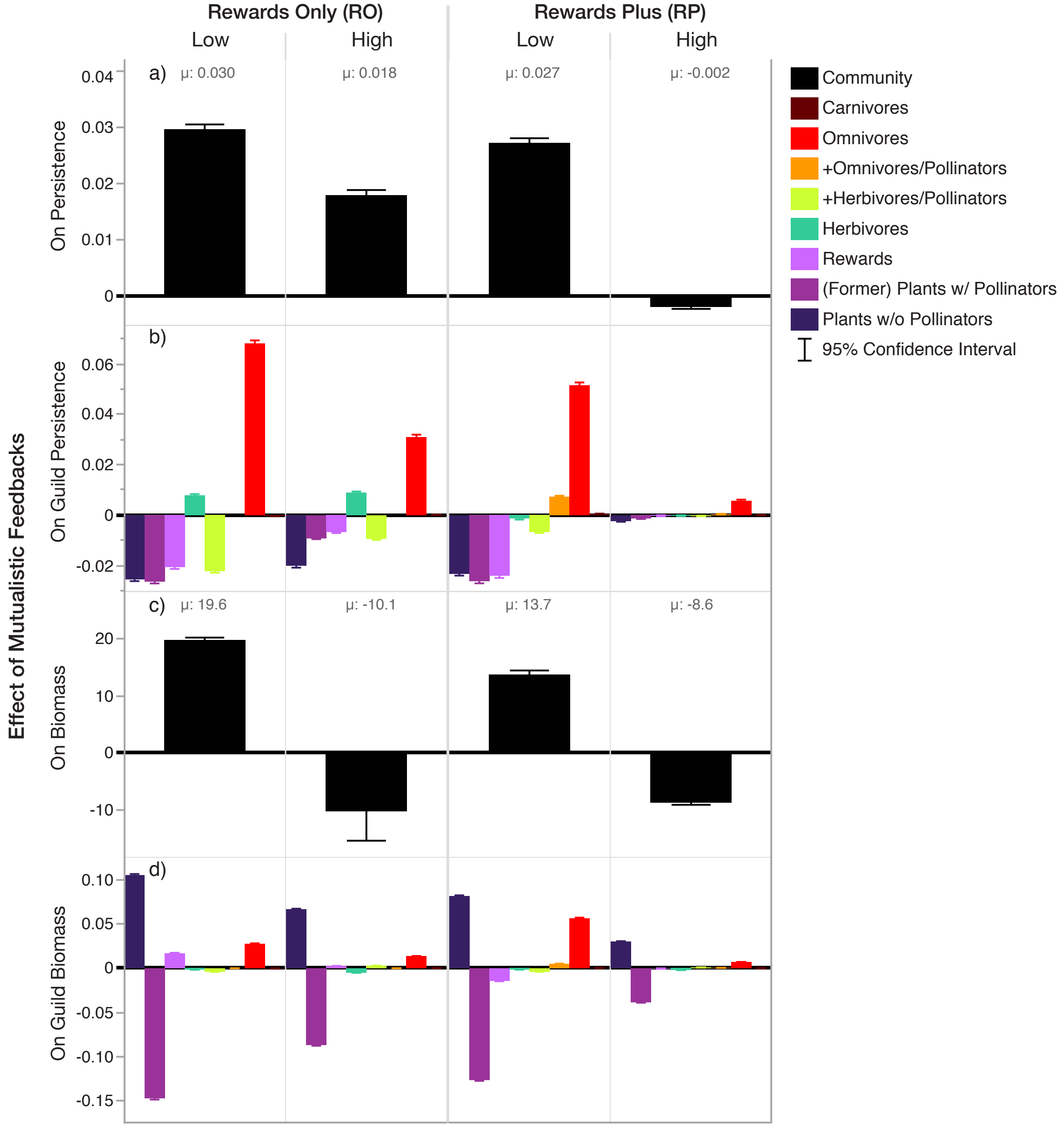

Absolute differences in steady-state a) persistence or c) total ecosystem biomass of the feedback control simulations compared to their paired multiplex simulations. Though changes in ecosystem persistence and biomass are trivial, feedback controls deviate systematically in the fraction of b) persisting species composed by each guild and d) total biomass composed by each guild. Positive values indicate positive effects of mutualistic feedbacks on persistence or biomass (corresponding to decreased persistence or biomass in controls compared to multiplex simulations). The black bold line shows zero change. Error bars are 95% confidence intervals around absolute differences averaged over the $N = 24{,}276$ simulations in each treatment (μ).

Supplementary Figure 9: Example timeseries comparing multiplex and feedback control simulations.

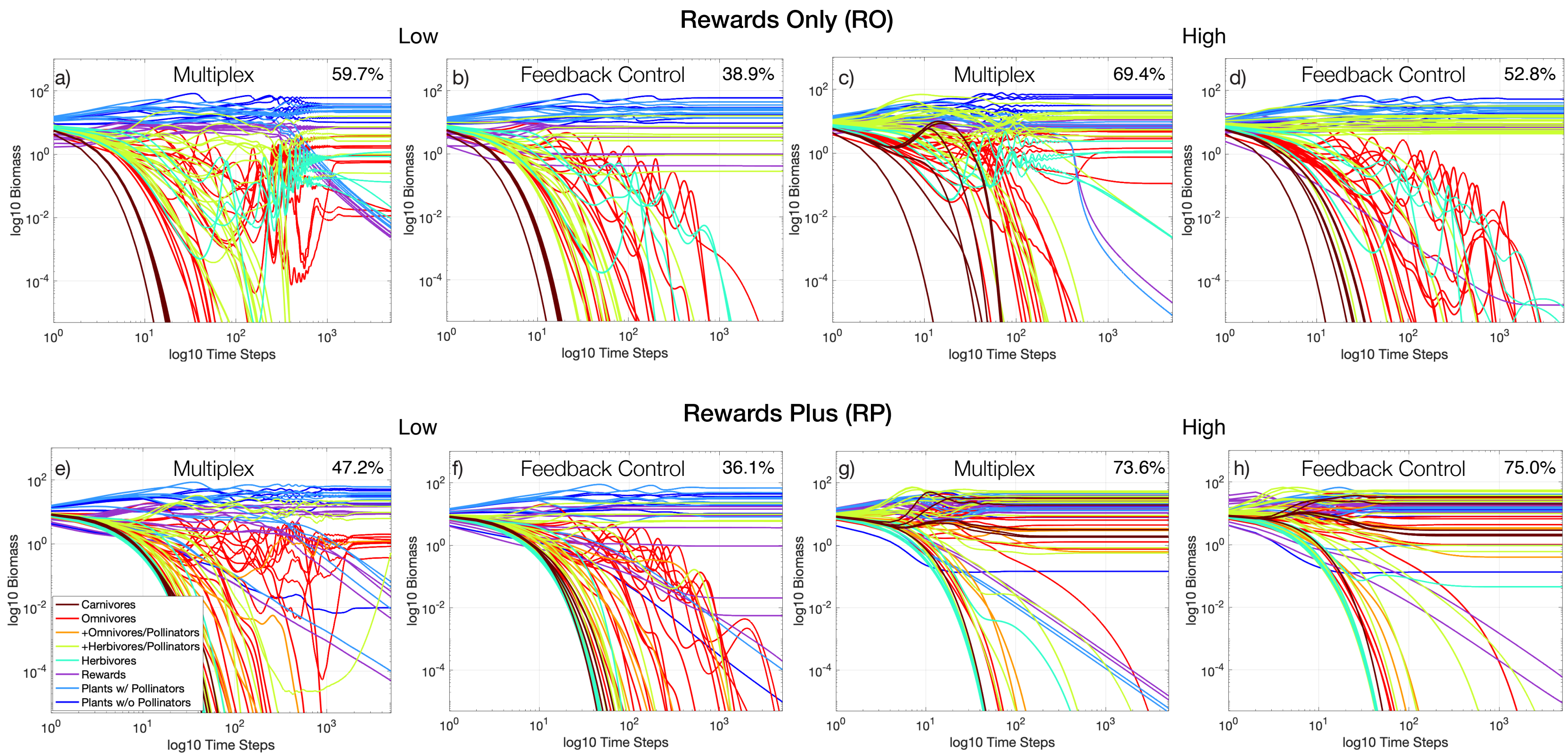

This example uses a 50-species niche-model food web integrated with a 33-species plant-pollinator network according to the Rewards Only (RO, a-d) and Rewards Plus (RP, e-h) network treatments subjected multiplex dynamics with Low (a-b, e-f) or High (c-d, g-h) rewards productivity. Note that 11 plant species in the food web are chosen to represent the 11 plant species in the pollination network. Multiplex simulations in each treatment (a, c, e, g) are compared to their paired feedback control simulations (c, d, f, h, respectively).

SUPPLEMENTARY REFERENCES

1. Cohen, J. E. & Briand, F. (1984). Trophic links of community food webs. *Proc. Natl. Acad. Sci. USA*, 81, 4105-4109.
2. Martinez, N. D. (1991). Artifacts or attributes? Effects of resolution on the Little Rock Lake food web. *Ecol. Monogr.*, 61, 367-392.
3. Williams, R.J. & Martinez, N.D. (2000). Simple rules yield complex food webs. *Nature*, 404, 180-183.
4. Dunne, J.A. (2006). The network structure of food webs, in *Ecological Networks Linking Structure to Dynamics in Food Webs*, pp. 27-86. Oxford U. Press, Oxford.
5. Valdovinos, F.S., Brosi, B.J., Briggs, H.M., Moisset de Espanés, P., Flores, J.D., Ramos-Jiliberto, R. & Martinez, N.D. (2016). Niche partitioning due to adaptive foraging reverses effects of nestedness and connectance on pollination network stability. *Ecol. Lett.*, 19, 1277-1286.
6. Thébault, E. & Fontaine, C. (2010). Stability of ecological communities and the architecture of mutualistic and trophic networks. *Science*, 329, 853-856.
7. Bascompte, J., Jordano, P., Melian, C.J. & Olesen, J.M. (2003). The nested assembly of plant-animal mutualistic networks. *Proc. Natl Acad. Sci. USA*, 100, 9383-9387.
8. Okuyama, T. & Holland, J.N. (2008). Network structural properties mediate the stability of mutualistic communities. *Ecol. Lett.*, 11, 208-216.
9. Allesina, S. & Tang, S. (2012). Stability criteria for complex ecosystems. *Nature*, 483, 205-208.
10. James, A., Pitchford, J.W. & Plank, M.J. (2012). Disentangling nestedness from models of ecological complexity. *Nature*, 487, 227-230.
11. Rohr, R.P., Saavedra, S. & Bascompte, J. (2014). On the structural stability of mutualistic systems. *Science*, 25, 416-425.
12. Pascual-García, A. & Bastolla, U. (2017) Mutualism supports biodiversity when the direct competition is weak. *Nat. Comm.*, 8:14326, 1-13.
13. Saavedra, S. & Stouffer, D.B. (2013). "Disentangling nestedness" disentangled. *Nature*, 500, E1-E2.
14. Guimarães, P. R. & Guimarães P. (2006). Improving the analyses of nestedness for large sets of matrices. *Environ. Modell. Software* **21**, 1512-1513.
15. Almeida-Neto M., Guimaraes P., Guimaraes P.R., Loyola R.D. & Ulrich W. 2008. A consistent metric for nestedness analysis in ecological systems: reconciling concept and measurement. Oikos 117: 1227-1239. https://www.guimaraes.bio.br/soft.html
16. Newman, M.E.J. (2006). Modularity and community structure in networks. *PNAS*, 103 (23), 8577-8582.
17. Flores, C.O., Poisot, T., Valverde, S., & Weitz, J.S. (2015) BiMat: A MATLAB package to facilitate the analysis of bipartite networks. *Methods in Ecol. Evol.*, 7. 127-132.
18. Berlow, E. L. *et al.* (2009) Simple prediction of interaction strengths in complex food webs. *Proc. Natl Acad. Sci. USA,* 106, 187–191.
19. Williams, R. J. & Martinez, N. D. (2004). Stabilization of chaotic and non-permanent food-web dynamics. *Eur. Phys. J. B,* 38, 297–303.
20. Martinez, N. D., Williams, R. J., & Dunne, J. A. (2006) Diversity, Complexity, and Persistence in Large Model Ecosystems, in *Ecological Networks Linking Structure to Dynamics in Food Webs*, pp. 163–184. Oxford U. Press, Oxford.
