# Supplementary material for: Mutualism increases diversity, stability, and function of multiplex networks that integrate pollinators into food webs
Source: Nat Commun. 2020 May 1;11:2182. doi: 10.1038/s41467-020-15688-w (PMC7195475; doi:10.1038/s41467-020-15688-w)
Supplement: Supplementary file 3 — Reporting Summary [file 41467_2020_15688_MOESM3_ESM.pdf]

nature research

Corresponding author(s): Kayla R. S. Hale

Last updated by author(s): March 10, 2020

Reporting Summary

Nature Research wishes to improve the reproducibility of the work that we publish. This form provides structure for consistency and transparency in reporting. For further information on Nature Research policies, see Authors & Referees and the Editorial Policy Checklist .

Statistics

For all statistical analyses, confirm that the following items are present in the figure legend, table legend, main text, or Methods section.

n/a	Confirmed	
☐	☒	The exact sample size (*n*) for each experimental group/condition, given as a discrete number and unit of measurement
☒	☐	A statement on whether measurements were taken from distinct samples or whether the same sample was measured repeatedly
☐	☒	The statistical test(s) used AND whether they are one- or two-sided<br>*Only common tests should be described solely by name; describe more complex techniques in the Methods section.*
☒	☐	A description of all covariates tested
☒	☐	A description of any assumptions or corrections, such as tests of normality and adjustment for multiple comparisons
☐	☒	A full description of the statistical parameters including central tendency (e.g. means) or other basic estimates (e.g. regression coefficient) AND variation (e.g. standard deviation) or associated estimates of uncertainty (e.g. confidence intervals)
☐	☐	For null hypothesis testing, the test statistic (e.g. *F*, *t*, *r*) with confidence intervals, effect sizes, degrees of freedom and *P* value noted<br>*Give P values as exact values whenever suitable.*
☒	☐	For Bayesian analysis, information on the choice of priors and Markov chain Monte Carlo settings
☒	☐	For hierarchical and complex designs, identification of the appropriate level for tests and full reporting of outcomes
☒	☐	Estimates of effect sizes (e.g. Cohen's *d*, Pearson's *r*), indicating how they were calculated

Our web collection on statistics for biologists contains articles on many of the points above.

Software and code

Policy information about availability of computer code

Data collection	Custom simulation code for network structures and dynamics was developed and run in MATLAB (The MathWorks 2018b). Dynamic simulations were run using MATLAB's R2018b Parallel Computing Toolbox with six cores. All custom code is publicly available at https://github.com/kayla-hale/Multiplex-Dynamics/. The ANINHADO software package used for analyzing nestedness of the plant-pollinator networks (Guimarães & Guimarães 2006, Almeida-Neto et al. 2008) is available at https://www.guimaraes.bio.br/soft.html.
Data analysis	Data analysis was performed in JMP 14 (SAS Institute 2018).

For manuscripts utilizing custom algorithms or software that are central to the research but not yet described in published literature, software must be made available to editors/reviewers. We strongly encourage code deposition in a community repository (e.g. GitHub). See the Nature Research guidelines for submitting code & software for further information.

Data

Policy information about availability of data

All manuscripts must include a data availability statement. This statement should provide the following information, where applicable:

- Accession codes, unique identifiers, or web links for publicly available datasets
- A list of figures that have associated raw data
- A description of any restrictions on data availability

Network structures and parameterization to reproduce Figs. 3-6 are available in the online repository at https://github.com/kayla-hale/Multiplex-Dynamics/.

Field-specific reporting

Please select the one below that is the best fit for your research. If you are not sure, read the appropriate sections before making your selection.

Ecological, evolutionary & environmental sciences study design

All studies must disclose on these points even when the disclosure is negative.

Study description	Custom code was developed to simulate stochastic network structures and deterministic network dynamics. The study used two treatments for network structure, corresponding to pollinator diet: Rewards Only (RO) and Rewards Plus (RP). N = 24,276 networks for each treatment were generated. The study used four treatments for network dynamics on these network structures: traditional Food Web (FW, with only feeding interactions) and Multiplex dynamics (including both reproductive and feeding interactions). Multiplex dynamics additionally were run with either the rewards productivity parameter set to either High (ß = 1) or Low (ß = 0.2). This resulted in six treatments - RO FW, RO Low, RO High, RP FW, RP Low, RP High - with N = 24,276 dynamical simulations for each.
Research sample	A single dynamical simulation corresponds to a single network structure (RO or RP) subjected to either FW or Multiplex dynamics (with either High or Low rewards productivity) for 5000 timesteps. Body masses for each species in a network were chosen randomly following previous work; metabolic parameters were calculated for each species from body mass using allometric relationships. All other parameters were fixed across treatments.
Sampling strategy	No sampling strategy was needed. All N = 24,276 dynamical simulations were used to calculate the summary statistics presented in the main text.
Data collection	KRSH developed all simulation code. KRSH ran and analyzed all simulations.
Timing and spatial scale	All main-text data was generated and analyzed in 2019 by KRSH.
Data exclusions	No data were excluded from analyses.
Reproducibility	Network dynamics are deterministic. Repeated trials of simulations using identical network structures and parameters but different simulation lengths or initial conditions result in qualitatively robust outcomes. Extensive analyses of the effect of variation in parameter values are presented in the SI.
Randomization	Network structures and dynamics were simulated following six treatments. No randomization of data during analyses was required.
Blinding	Blinding was not relevant to this structure. Network structures and dynamics were simulated following six treatments. KRSH analyzed representative timeseries to understand the interplay of network structure and dynamics on resulting diversity, stability, and function.

Did the study involve field work? ☐ Yes ☒ No

Reporting for specific materials, systems and methods

We require information from authors about some types of materials, experimental systems and methods used in many studies. Here, indicate whether each material, system or method listed is relevant to your study. If you are not sure if a list item applies to your research, read the appropriate section before selecting a response.

Materials & experimental systems

n/a	Involved in the study
☒	☐ Antibodies
☒	☐ Eukaryotic cell lines
☒	☐ Palaeontology
☒	☐ Animals and other organisms
☒	☐ Human research participants
☒	☐ Clinical data

Methods

n/a	Involved in the study
☒	☐ ChIP-seq
☒	☐ Flow cytometry
☒	☐ MRI-based neuroimaging
